# The Genus *Gnaphalium* L. (Compositae): Phytochemical and Pharmacological Characteristics

**DOI:** 10.3390/molecules18078298

**Published:** 2013-07-15

**Authors:** Xing Zheng, Wei Wang, Huishan Piao, Weiqiang Xu, Haibo Shi, Chengai Zhao

**Affiliations:** 1 College of Pharmacy, Yanbian University, Yanji 133002, Jilin, China; E-Mail: zhengxing@ybu.edu.cn; 2 Institute of Phytochemistry, Jilin Academy of Chinese Medicine Sciences, Changchun 130012, Jilin, China; E-Mail: shihaibo3901@163.com; 3 College of Resources and Environment, Jilin Agricultural University, Changchun 130118, Jilin, China; E-Mails: xuweiqiang198811@163.com (W.X.); zca136@163.com (C.Z.)

**Keywords:** *Gnaphalium*, chemical constituents, biological activities

## Abstract

The genus *Gnaphalium*, a herb distributed worldwide, comprises approximately 200 species of the Compositae (Asteraceae) family that belongs to the tribe Gnaphalieae. Some species are traditionally used as wild vegetables and in folk medicine. This review focuses on the phytochemical investigations and biological studies of plants from the genus *Gnaphalium* over the past few decades. More than 125 chemical constituents have been isolated from the genus *Gnaphalium*, including flavonoids, sesquiterpenes, diterpenes, triterpenes, phytosterols, anthraquinones, caffeoylquinic acid derivatives, and other compounds. The extracts of this genus, as well as compounds isolated from it, have been demonstrated to possess multiple pharmacological activities such as antioxidant, antibacterial and antifungal, anti-complement, antitussive and expectorant, insect antifeedant, cytotoxic, anti-inflammatory, antidiabetic and antihypouricemic properties. The present review compiles the information available on this genus because of its relevance to food and ethnopharmacology and the potential therapeutic uses of these species.

## 1. Introduction

The genus *Gnaphalium*, a variable annual or perennial herb distributed worldwide, comprises approximately 200 species of the Compositae (Asteraceae) family that belongs to the tribe Gnaphalieae. Among them, 19 species are widespread in the Yangtze and Pearl river basins of China [1]. *G*. *affine* is an annual herbaceous plant that grows widely in East Asia, including the temperate regions of China, Korea, and Japan as well as some high altitude tropical regions of India, Nepal, and Thailand. The species is traditionally used as a wild vegetable in the Guangdong and Fujian provinces of China [2,3]. Every year after the traditional Ching Ming festival, it is extensively harvested nationally as a wild vegetable and then processed into a variety of foods, such as drinks, canned products, and frozen vegetables. *G*. *affine* is believed to be of high nutritional value since it has a reasonable proportion of the eight essential amino acids for human body, a high content of minerals, trace elements, and vitamins, and is thus considered suitable to be developed into a functional food [4]. *G*. *polycaulon* is an annual widespread weed in tropical and subtropical Africa, Asia, Australia, and America. The aerial parts are available in the cool season from November to January and are used as a flavor ingredient in foods for carminative purpose during the Chinese New Year celebrations by ethnic Chinese [5]. Besides the nutritional value, *G*. *affine* is used in traditional Chinese medicine for resolving phlegm, relieving cough, and dispelling wind-cold syndrome [6]. Traditional medical practitioners residing in the Balkan peninsula, Southeast Europe, use the aerial parts of *G*. *uliginosum* for the treatment of hypertension and ulcers. There is some information about the usage of the herb *G*. *uliginosum* for the treatment of the thromophlebitis and phlebothrombosis in Russian phytotherapy [7,8]. Some species of the genus *Gnaphalium*, commonly known as “gordolobo”, are used as folk medicine in Mexico to treat various respiratory diseases, such as grippe, fever, asthma, cough, cold, bronchitis, expectorating, and bronchial affections [9]. In some Latin American countries plants belonging to the genus *Gnaphalium* are traditionally used for the relief of stomach diseases, swelling, wounds, prostatism, lumbago, neuritis, and angina ache, for the lowering of blood pressure, or as diuretic, antipyretic, and antimalarial [10]. *G*. *pellitum* is used as an ornamental plant and applied to reduce swelling in German folk medicine [11]. The flower of *G*. *stramineum*, a Central-American herbaceous plant commonly known as “sanalotodo”, is used in traditional medicine as anti-inflammatory and anti-rheumatic agents [12]. *G*. *graveolens* is used to cure skin infections and as an anti-inflammatory agent, and in certain regions it is also used against cancer [13]. The inflorescence of *G*. *sylvaticum* is used in Polish folk medicine as a diuretic and vermifuge [14]. The leaves of *G. luteo-album* are used as astringent, cholagogue, diuretic, febrifuge, and haemostatic [15]. In addition, some Gnaphalium species are used in poultices to tend wounds, as a hemostatic, to fight infections, or ease inflammation. In the Andean regions of South America, the hot beverage obtained by decoction of *G*. *purpureum* and *G*. *elegans* is recommended for the cure of cancer [16].

Previous phytochemical investigations of the genus have led to the identification of *ca*. 125 chemical constituents in total, including flavonoids, sesquiterpenes, diterpenes, triterpenes, phytosterols, anthraquinones, caffeoylquinic acid derivatives, and other compounds. Biological studies have demonstrated antioxidant, antibacterial and antifungal, anti-complement, antitussive and expectorant, insect antifeedant, cytotoxic, anti-inflammatory, antidiabetic, antihypouricemic, and other activities of the extracts and chemical constituents of *Gnaphalium* species. To the best of our knowledge, there have not been any in-depth reviews on this genus from the phytochemical and biological viewpoints. Here, we compile the phytochemical and biological researches on the genus *Gnaphalium* during the past few decades.

## 2. Chemical Constituents

During the past decades, more than 125 secondary metabolites were isolated and identified from species of *Gnaphalium*. Here, the structures of 68 flavonoids, two sesquiterpenes, 28 diterpenes, five triterpenes, four phytosterols, two anthraquinones, five caffeoylquinic acid derivatives, and 10 other compounds are shown below, of which the names, corresponding plant sources, and references are collected in Table 1.

**Table 1:** Flavonoids, sesquiterpenes, diterpenes, triterpenes, phytosterols, anthraquinones, caffeoylquinic acid derivatives, and other compounds from the genus *Gnaphalium*.

No.	Compound	Source	Ref.
**Flavonoids**			
**1**	Apigenin	*G*. *affine*	[17]
		*G*. *hypoleucum*	[18]
		*G*. *luteo-album*	[19]
		*G*. *sylvaticum*	[20]
**2**	Apigenin 4'-*O*-*β*-d-glucopyranoside	*G*. *affine*	[21]
**3**	Apigenin 7-*O*-*β*-d-glucopyranoside	*G*. *luteo-album*	[19]
		*G*. *uliginosum*	[22]
**4**	Apigenin 4'-*O*-*β*-d-(6''-*E*-caffeoyl)-glucopyranoside	*G*. *affine*	[17]
**5**	Apigenin 7-*O*-*β*-d-(6''-*E*-caffeoyl)-glucopyranoside	*G*. *affine*	[17]
**6**	Luteolin	*G*. *affine*	[17]
		*G*. *hypoleucum*	[18]
		*G*. *indicum*	[23]
		*G*. *luteo-album*	[19]
		*G*. *rufescens*	[24]
		*G*. *sylvaticum*	[20]
**7**	Luteolin 4'-*O*-*β*-d-glucopyranoside	*G.* *affine*	[17]
		*G*. *cheiranthifolium*	[25]
		*G*. *hypoleucum*	[18]
		*G*. *luteo-album*	[19]
**8**	Luteolin 7-*O*-*β*-d-glucopyranoside	*G*. *luteo-album*	[19]
**9**	Luteolin 4'-*O*-*β*-d-(6''-*E*-caffeoyl)-glucopyranoside	*G*. *affine*	[17]
**10**	6-Hydroxyluteolin 7-*O*-*β*-d-glucopyranoside	*G*. *affine*	[17]
		*G*. *uliginosum*	[26]
**11**	6-Methoxyluteolin	*G*. *uliginosum*	[26]
**12**	Luteolin 7-*O*-methyl ether	*G*. *rufescens*	[24]
**13**	5,7,3',4'-Tetrahydroxy-6-methoxyflavone 7-*O*-*β*-d-glucopyranoside	*G*. *uliginosum*	[22]
**14**	5,7,3',4'-Tetrahydroxy-6-methoxyflavone 7-*O*-*β*-d-(6''-*E-*caffeoyl)-glucopyranoside	*G*. *uliginosum*	[26]
**15**	Scutellarein 7-*O*-β-d-glucopyranoside	*G*. *uliginosum*	[26]
**16**	Acacetin 7-*O*-rutinoside	*G*. *affine*	[17]
**17**	5-Hydroxy-7,8-dimethoxyflavone	*G*. *pellitum*	[27]
**18**	5-Hydroxy-4',7-dimethoxyflavone	*G*. *affine*	[17]
**19**	Velutin	*G*. *gaudichaudianum*	[28]
**20**	5,8-Dihydroxy-6,7-dimethoxyflavone	*G*. *gaudichaudianum*	[29]
**21**	Hispidulin	*G*. *antennarioides*	[24]
**22**	Hispidulin 7-*O*-*β*-d-glucopyranoside	*G*. *antennarioides*	[24]
**23**	Tricin	*G*. *sylvaticum*	[20]
**24**	Jaceosidin	*G*. *luteo-album*	[19]
		*G*. *uliginosum*	[30]
**25**	8-*O*-(2-Methylbutyryl)-5,7,8-trihydroxyflavone	*G*. *robuscum*	[31]
**26**	8-*O*-[(*Z*)-2-Methyl-2-butenoyl]-5,7,8-trihydroxyflavone	*G*. *robuscum*	[31]
**27**	5,7,4'-Trihydroxy-3'-methoxyflavone 7-*O*-*β*-d-glucopyranoside	*G*. *uliginosum*	[22]
**28**	Gnaphaloside A	*G*. *uliginosum*	[22]
**29**	Kaempferol	*G*. *affine*	[17]
		*G*. *uniflorum*	[32]
**30**	Isokaempferide	*G*. *dioicum*	[33]
**31**	Quercetin	*G*. *affine*	[17]
		*G*. *gracile*	[34]
		*G*. *hypoleucum*	[18]
		*G*. *indicum*	[23]
		*G*. *pellitum*	[35]
		*G*. *sylvaticum*	[20]
		*G*. *uniflorum*	[32]
**32**	Quercetin 4'-*O*-*β*-d-glucopyranoside	*G*. *affine*	[17]
		*G*. *hypoleucum*	[18]
**33**	Quercetin 4'-*O*-*β*-d-(6''-*E*-caffeoyl)-glucopyranoside	*G*. *affine*	[17]
**34**	Quercetin 7-*O*-*β*-d-glucuronide	*G*. *affine*	[17]
**35**	Quercimeritrin	*G*. *affine*	[17]
		*G*. *sylvaticum*	[20]
**36**	Isoquercitrin	*G*. *stramineum*	[36]
		*G*. *sylvaticum*	[20]
		*G*. *uliginosum*	[22]
		*G*. *uniflorum*	[32]
**37**	Quercetin 3-*O*-*β*-d-galactopyranoside	*G*. *stramineum*	[36]
**38**	Rutin	*G*. *stramineum*	[36]
		*G*. *uniflorum*	[32]
**39**	Quercetin 3-*O*-*β*-d-galactopyranoside-4'-*O*-*β*-d-glucopyranoside	*G*. *uniflorum*	[32]
**40**	Quercetagetin	*G*. *affine*	[17]
**41**	Quercetagetin 7-*O*-*β*-d-glucopyranoside	*G*. *affine*	[17]
**42**	Isorhamnetin	*G*. *affine*	[17]
**43**	Isorhamnetin 3-*O*-*β*-d-galactopyranoside	*G*. *uniflorum*	[32]
**44**	3,5,7,4'-Tetrahydroxy-3'-methoxyflavone 3-*O*-*β*-d-glucopyranoside	*G*. *uliginosum*	[22]
**45**	3,5,7,3',4'-Pentahydroxy-6-methoxyflavone 3-*O*-*β*-d-glucopyranoside	*G*. *uliginosum*	[22]
**46**	Gnaphaliin B	*G*. *affine*	[17]
		*G*. *liebmannii*	[37]
**47**	3,5-Dihydroxy-6,7,8-trimethoxyflavone	*G*. *chilense*	[38]
		*G*. *microecephalum*	[38]
		*G*. *robustum*	[39]
**48**	3,5-Dihydroxy-6,7,8,4'-tetramethoxyflavone	*G*. *affine*	[17]
**49**	5-Hydroxy-3,7,8-trimethoxyflavone	*G*. *affine*	[17]
		*G*. *robustum*	[39]
		*G*. *obtusifolium*	[40]
**50**	5-Hydroxy-3,6,7,8-tetramethoxyflavone	*G*. *affine*	[41]
		*G*. *hypoleucum*	[18]
		*G*. *undulatum*	[42]
**51**	5-Hydroxy-3,6,7,8,4'-pentamethoxyflavone	*G*. *affine*	[17]
		*G*. *hypoleucum*	[18]
**52**	5-Hydroxy-3,6,7,8,3',4'-hexamethoxyflavone	*G*. *affine*	[17]
		*G*. *hypoleucum*	[18]
**53**	Gnaphaliin A	*G*. *affine*	[17]
		*G*. *gracile*	[34]
		*G*. *lanuginosum*	[43]
		*G*. l*iebmannii*	[37]
		*G*. *obtusifolium*	[40]
		*G*. *robustum*	[39]
**54**	5,7-Dihydroxy-3-methoxyflavone	*G*. *gracile*	[34]
		*G*. *robustum*	[39]
**55**	8-*O*-(2-Methyl-2-butenoyl)-5,7-dihydroxy-3-methoxyflavone	*G*. *robustum*	[44]
**56**	5,7-Dihydroxy-3,6-dimethoxyflavone	*G*. *wrightii*	[42]
**57**	5,8-Dihydroxy-3,6,7-trimethoxyflavone	*G*. *gaudichaudianum*	[29]
**58**	5,7-Dihydroxy-3,6,8-trimethoxyflavone	*G*. *affine*	[17]
		*G*. *elegans*	[16]
**59**	5,7-Dihydroxy-3,8,4'-trimethoxyflavone	*G*. *affine*	[17]
**60**	5,7-Dihydroxy-3,8,3',4'-tetramethoxyflavone	*G*. *affine*	[17]
**61**	5,6-Dihydroxy-3,7-dimethoxyflavone	*G*. *affine*	[41]
**62**	3,5,7-Trihydroxy-6,8-dimethoxyflavone	*G*. *obtusifolium*	[45]
**63**	5,7,8-Trihydroxy-3-methoxyflavone	*G*. *robuscum*	[39]
**64**	Quercetin 3-methyl ether	*G*. *gracile*	[34]
		*G*. *indicum*	[23]
**65**	Rhamnetin	*G*. *pellitum*	[35]
**66**	Pinocembrin	*G*. *purpurascens*	[46]
**67**	4,4',6'-Trihydroxy-2'-methoxychalcone	*G*. *affine*	[41]
**68**	Gnaphalin	*G*. *affine*	[47]
		*G*. *cheiranthifolium*	[25]
		*G*. *multiceps*	[48]
		*G*. *purpurascens*	[46]
		*G*. *luteo-album*	[19]
	**Sesquiterpenes**		
**69**	Germacrene D	*G*. *oligandrum*	[42]
**70**	(2*E*,6*Z*)-7,11,11-trimethylbicyclo[8.1.0]undeca-2,6-diene	*G*. *oligandrum*	[42]
	Dit erpenes		
**71**	Sclareol	*G*. *gaudichaudianum*	[49]
**72**	8*α*,13*α*-Diacetoxysclareol	*G*. *gaudichaudianum*	[49]
**73**	8-*epi*-Sclareol	*G*. *undulatum*	[42]
**74**	13-*epi*-Sclareol	*G*. *pellitum*	[11]
		*G*. *graveolens*	[11]
**75**	13-*epi*-Cyclosclareol	*G*. *pellitum*	[11]
		*G*. *graveolens*	[11]
		*G*. *undulatum*	[42]
**76**	Kauranol	*G*. *rufescens*	[24]
**77**	Kaur-16-en-19-oic acid	*G*. *gaudichaudianum*	[49]
		*G*. *inornatum*	[46]
		*G*. *rufescens*	[24]
**78**	Methyl kaur-16-en-19-oate	*G*. *gaudichaudianum*	[49]
**79**	3*α*-Hydroxykaur-16-en-19-oic acid	*G*. *gaudichaudianum*	[49]
**80**	Methyl 3*α*-hydroxykaur-16-en-19-oate	*G*. *gaudichaudianum*	[49]
**81**	11*β*-Acetoxykaur-16-en-19-oic acid	*G*. *rufescens*	[24]
**82**	3*α*-Acetoxykaur-16-en-19-oic acid	*G*. *gaudichaudianum*	[49]
**83**	Methyl 3*α*-acetoxykaur-16-en-19-oate	*G*. *gaudichaudianum*	[49]
**84**	*ent*-Kauran-16-ene	*G*. *undulatum*	[42]
**85**	*ent*-Kaur-16-en-19-al	*G*. *undulatum*	[42]
**86**	*ent*-Kaur-16-en-19-oic acid	*G*. *graveolens*	[11]
		*G*. *oligandrum*	[42]
		*G*. *pellitum*	[11]
		*G*. *undulatum*	[42]
**87**	15*α*-Hydroxy-*ent*-kaur-16-en-19-oic acid	*G*. *undulatum*	[42]
**88**	11*β*-Acetoxy-*ent*-kaur-16-en-19-oic acid	*G*. *pellitum*	[11]
**89**	*ent*-Kaur-9(11),16-dien-19-oic acid	*G*. *oligandrum*	[42]
		*G*. *undulatum*	[42]
**90**	Sylviside	*G*. *sylvaticum*	[20]
**91**	*ent*-Pimar-15-ene-3*α*,8*α*-diol	*G*. *gaudichaudianum*	[28]
**92**	*ent*-Pimar-15-ene-8*α*,19-diol	*G*. *gaudichaudianum*	[28]
**93**	*ent*-Pimara-8(14),15-dien-3*α*-ol	*G*. *gaudichaudianum*	[28]
**94**	*ent*-Pimara-8(14),15-dien-19-ol	*G*. *gaudichaudianum*	[28]
**95**	*ent-*Pimara-8(14),15-dien-3*α*,19-diol	*G*. *gaudichaudianum*	[28]
**96**	*ent*-Pimara-8(14),15-dien-19-oic acid	*G*. *gaudichaudianum*	[28]
**97**	*ent*-Pimara-8(14),15-dien-18-oic acid	*G*. *gaudichaudianum*	[28]
**98**	15*β*-hydroxy-wedeliaseccokaurenolide	*G*. *undulatum*	[50]
**Triterpenes**			
**99**	*α*-Amyrin	*G*. *affine*	[51]
**100**	Taraxasterol acetate	*G*. *affine*	[51]
**101**	*β*-Amyrin	*G*. *affine*	[51]
**102**	Betulinic acid	*G*. *affine*	[51]
**103**	Squalene	*G*. *gaudichaudianum*	[28]
**Phytosterols**			
**104**	β-Sitosterol	*G*. *affine*	[51]
		*G*. *hypoleucum*	[18]
		*G*. *inornatum*	[46]
		*G*. *pellitum*	[27]
**105**	(20*R*)-Cholest-4-en-3-on	*G*. *affine*	[51]
**106**	3*β*-Hydroxy-stigmast-5,22-dien-7-one	*G*. *affine*	[51]
**107**	Stigmasterol	*G*. *gaudichaudianum*	[28]
	**Anthraquinones**		
**108**	Emodin	*G*. *affine*	[51]
**109**	Physcion	*G*. *affine*	[51]
	**Caffeoylquinic acid derivatives**		
**110**	Chlorogenic acid	*G*. *uliginosum*	[22]
**111**	4-*O*-caffeoylquinic acid	*G*. *stramineum*	[36]
**112**	Cynarin	*G*. *uliginosum*	[22]
**113**	3,5-di-*O*-Caffeoylquinic acid	*G*. *stramineum*	[36]
**114**	4,5-di-*O*-Caffeoylquinic acid	*G*. *stramineum*	[36]
**115**	3,4,5-tri-*O*-Caffeoylquinic acid	*G*. *stramineum*	[36]
	**Other compounds**		
**116**	Gnaphaliol 3-*O*-*β-*d-glucopyranoside	*G*. *polycaulon*	[5]
**117**	Gnaphaliol 9-*O*-*β-*d-glucopyranoside	*G*. *polycaulon*	[5]
			
**118**	(*Z*)-3-Hexenyl *O*-β-d-glucopyranoside	*G*. *polycaulon*	[5]
**119**	Adenosine	*G*. *polycaulon*	[5]
**120**	Obliquine	*G*. *sphacelatum*	[46]
**121**	Scopoletin	*G*. *affine*	[47]
**122**	(+)-Pinitol	*G*. *pellitum*	[27]
**123**	Trans-caffeic acid	*G*. *uliginosum*	[22]
**124**	*n*-Tetracosanic acid	*G*. *hypoleucum*	[18]
**125**	*n*-Hexacosanic acid	*G*. *affine*	[51]

### 2.1. Flavonoids

**Figure 1:**
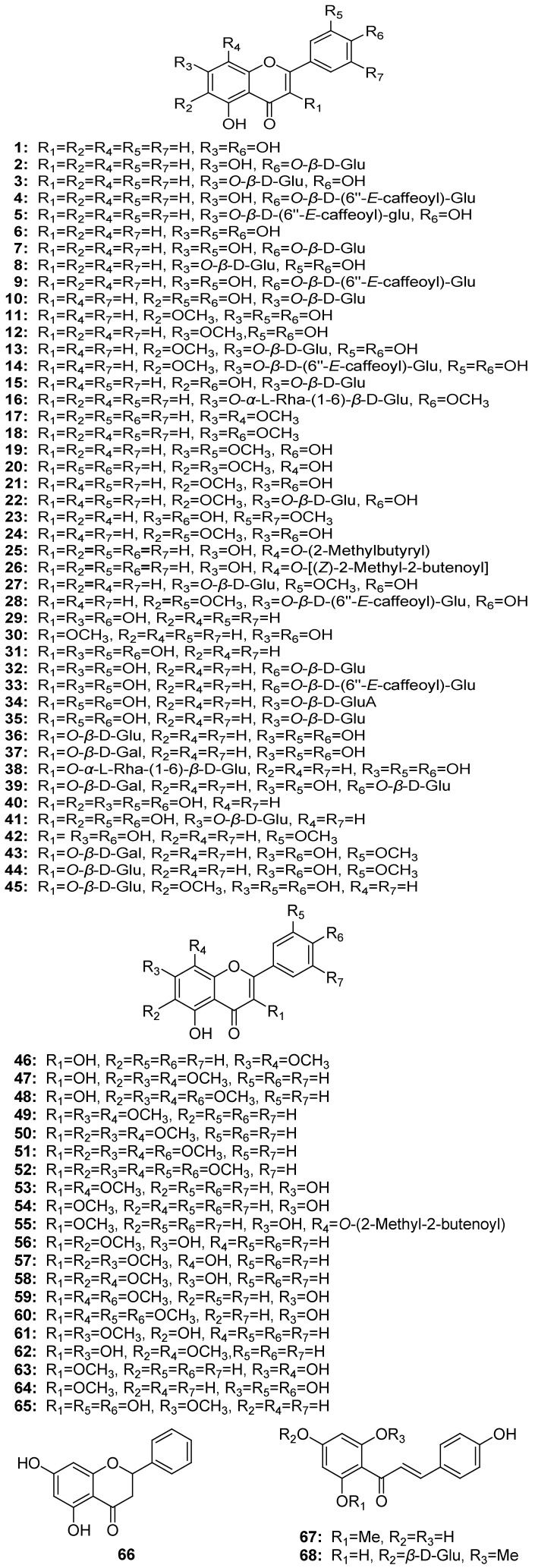
Chemical structures of flavonoids isolated from the genus *Gnaphalium*.

To date, 28 flavone derivatives, **1**–**28**, 37 flavonol derivatives, **29**–**65**, one flavanone, **66**, and two chalcone derivatives, **67**, **68**, have been reported, which constitute the majority of the secondary metabolites from the genus *Gnaphalium* [17,18,19,20,21,22,23,24,25,26,27,28,29,30,31,32,33,34,35,36,37,38,39,40,41,42,43,44,45,46,47,48]. Among them, **28** compounds, **2**–**5**, **7**–**10**, **13**–**16**, **23**, **27**, **28**, **32**–**39**, **41**, **43**–**45**, and **68**, were isolated as *O*-glycosides. The most frequently encountered flavonoids are quercetin (**31**), present in seven species, luteolin (**6**) and gnaphaliin A (**53**) found in six species, and ganphalin (**68**), which has been isolated from five species. Also apigenin (**1**), luteolin 4'-*O*-*β*-d-glucopyranoside (**7**), and isoquercitrin (**36**), were relatively common. The presence of gnaphaliin A (=5,7-dihydroxy-3,8-dimethoxyflavone, **53**), gnaphaliin B (=3,5-hydroxy-7,8-dimethoxyflavone, **46**), and gnalialin (=4,2',4'-trihydroxy-6'-methoxychalcone 4'-*β*-d-glucopyranoside), appear to be a characteristic chemotaxonomic feature typical of the genus *Gnaphalium* (Figure 1).

### 2.2. Sesquiterpenes

**Figure 2:**
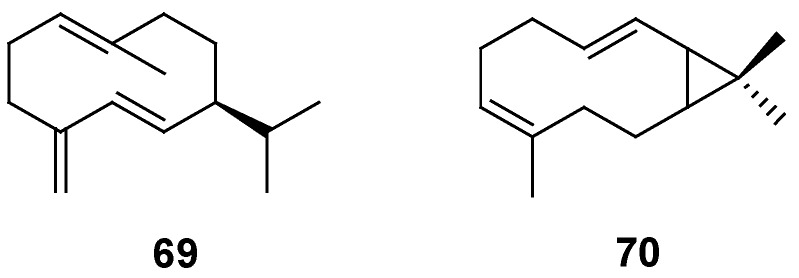
Chemical structures of sesquiterpenes isolated from the genus *Gnaphalium*.

In 1980, germacrene D (**69**) and (2*E*,6*Z*)-7,11,11-trimethylbicyclo[8.1.0]undeca-2,6-diene (**70**) were isolated from *G*. *oligandrum* [42] (Figure 2).

### 2.3. Diterpenes

**Figure 3:**
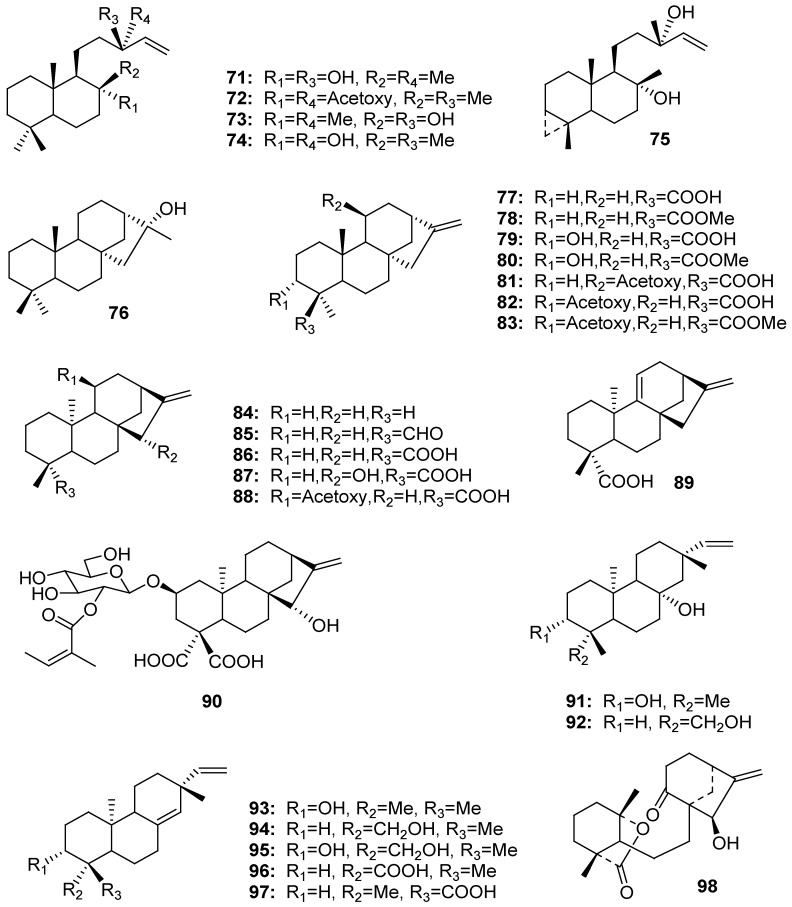
Chemical structures of diterpenes isolated from the genus *Gnaphalium*.

Five labdane-type diterpenes, **71**–**75**, were isolated from *G*. *gaudichaudianum*, *G*. *graveolens*, *G*. *pellitum*, and *G*. *undulatum* [11,49]. Approximately 15 kaurane-type diterpenes, **76**–**90**, were isolated, three, *i*.*e*., **76**, **77** and **81**, from *G*. *rufescens* [24], six, *i*.*e*., **77**, **78**–**80**, **82**, and **83**, from *G*. *gaudichaudianum* [49], one, *i*.*e*., **77**, from *G*. *inornatum* [46], five, *i*.*e*., **84**–**87** and **89**, from *G*. *undulatum* [42], one,* i*.*e*., **86**, from *G*. *graveolens* [11], two, *i*.*e*., **86** and **89**, from *G*. *oligandrum* [42], two, *i*.*e*., **86** and **88**, from *G*. *pellitum* [11], and sylviside (**90**) from *G*. *sylvaticum* [20]. Seven pimara-type diterpenes, **91**–**97**, from *G*. *gaudichaudianum* were isolated and identified [28]. Only one wedeliaseccokaurenolide derivative was characterized from *G*. *undulatum* [50] (Figure 3).

### 2.4. Tritepenes

**Figure 4:**
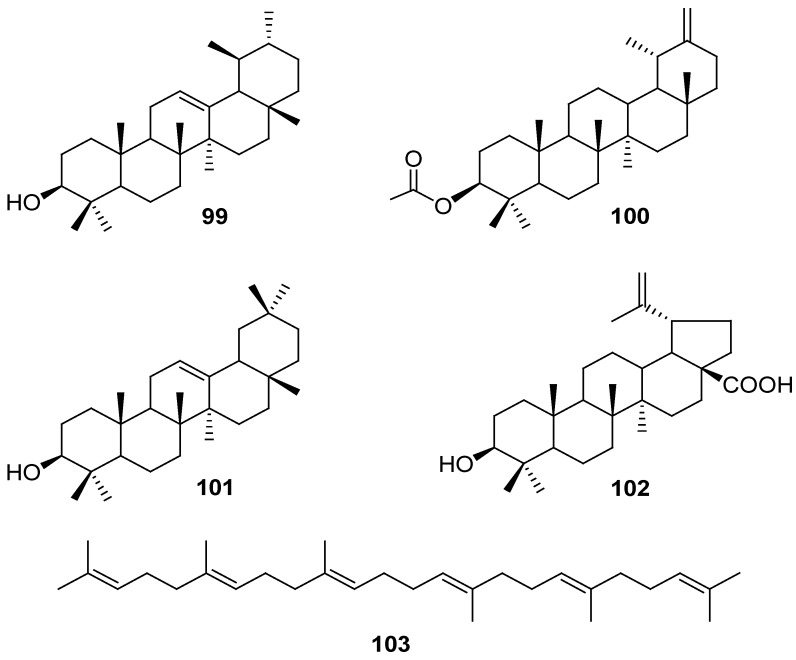
Chemical structures of triterpenes isolated from the genus *Gnaphalium*.

*α*-Amyrin, taraxasterol acetate, *β*-amyrin, and betulinic acid (**99**–**102**, resp.) were isolated from *G*. *affine* [51], and squalene (**103**) was identified from *G*. *gaudichaudianum* [28] (Figure 4).

### 2.5. Phytosterols

**Figure 5:**
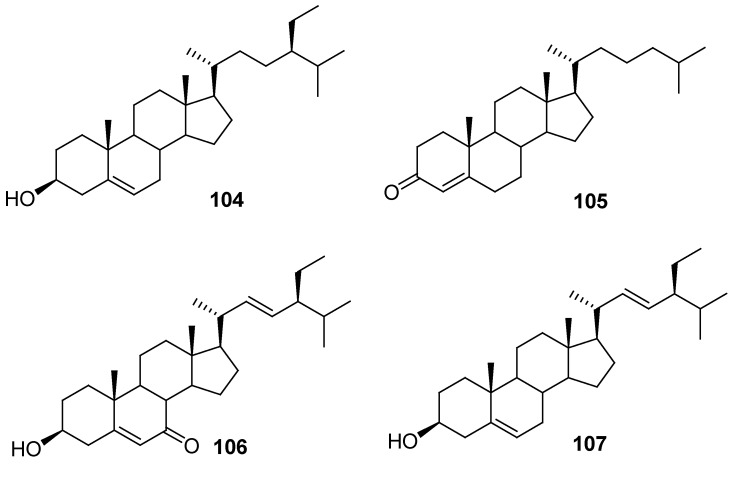
Chemical structures of phytosterols isolated from the genus *Gnaphalium*.

*β*-Sitosterol (**104**) was isolated from *G*. *affine*, *G*. *hypoleucum*, *G*. *inornatum*, and *G*. *pellitum* [18,27,46,51], and (20*R*)-cholest-4-en-3-one (**105**) and 3*β*-hydroxy-stigmast-5,22-dien-7-one (**106**) were also identified from *G*. *affine* [51]. Compound **107** belongs to stigmastane-type was obtained from *G*. *gaudichaudianum* [28] (Figure 5).

### 2.6. Anthraquinones

**Figure 6:**
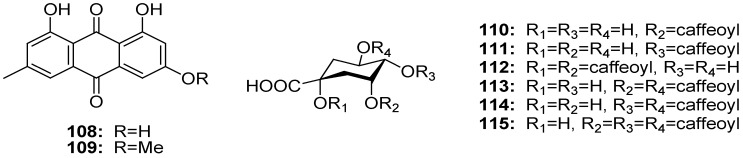
Chemical structures of anthraquinones and caffeoylquinic acid derivatives isolated from the genus *Gnaphalium*.

During the studies on chemical constituents of the petroleum ether fraction from *G. affine*, two anthraquinones (**108** and **109**) were obtained [51] (Figure 6).

### 2.7. Caffeoylquinic Acid Derivatives

Two caffeoylquinic acid derivatives, *i*.*e*., chlorogenic acid (**110**) and cynarin (**112**), were identified from the aerial parts of *G. uliginosum* [22], and four caffeoylquinic acid derivatives, *i*.*e*., 4-*O*-caffeoylquinic acid (**111**), 3,5-dicaffeoylquinic acid (**113**), 4,5-dicaffeoylquinic acid (**114**), and 3,4,5-tri-*O*-caffeoylquinic acid (**115**) were isolated from the flowers of *G*. *stramineum* [36] (Figure 6).

### 2.8. Other Compounds

**Figure 7:**
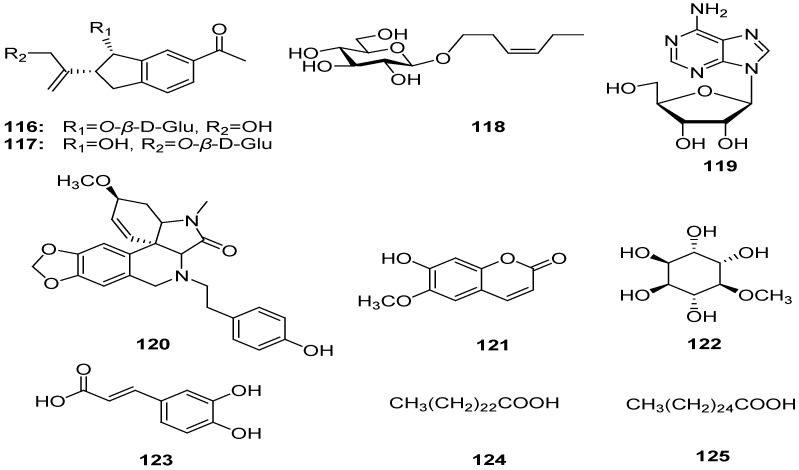
Chemical structures of other compounds isolated from the genus *Gnaphalium*.

A new 3-hydroxydihydrobenzofuran glucoside, gnaphaliol 9-*O*-*β*-d-glucopyranoside (**117**), was isolated from the aerial parts of *G. polycaulon* along with gnaphaliol 3-*O*-*β*-d-glucopyranoside (**116**), (*Z*)-3-hexenyl *O*-*β*-d-glucopyranoside (**118**), and adenosine (**119**) [5]. Besides the above-mentioned chemical constituents, some other compounds, **120**-**125**, also were isolated from this genus, their names and sources are compiled in the Table 1 [18,22,27,28,46,47,51] (Figure 7).

## 3. Biological Activities

### 3.1. Antioxidant Activity

The essential oil from *G. affine* was observed to possess strong radical scavenging activity against 2,2'-azinobis-3-ethylbenzthiazoline-6-sulfonate with the IC_50_ being 0.32 ± 0.89 μg/mL (IC_50_ of ascorbic acid = 24.06 ± 0.73 μg/mL). A significant inhibitory effect of the essential oil on lipid peroxidation in egg yolk homogenates was shown with an IC_50_ value of 0.09 ± 0.75 μg/mL (IC_50_ of ascorbic acid = 6.73 ± 0.87 μg/mL). The essential oil had a stronger reducing power than Vc in the reducing of Fe^3+^ to Fe^2+^ by donating an electron [52]. The scavenging effect elicited by the ethanolic extract of leaves of *G. uniflorum* was concentration-dependent in 2,2-diphenyl-1-picrylhydrazyl test, the amount of antioxidant necessary to decrease the initial concentration by 50% (SC_50_) was calculated as 15.96 μg of extract. The extract showed a concentration-dependent antioxidant activity also in the LP-LUV test (IC_50_ = 14.86 μg of extract), determination of the accumulation of products of peroxidation in mixed dipalmitoylphosphatidylcholine/linoleic acid unilamellar vesicles induced by the water-soluble peroxyl radical generator 2,2'-azobis(2-amidinopropane)hydrochloride [32].

### 3.2. Antibacterial and Antifungal Activity

The chloroform extract from *G*. *americanum*l possessed marked antimicrobial activity against the yeast tested *Candida albicans* with the minimum inhibitory concentration (MIC) being 5 mg/mL. The chloroform extracts from *G*. *oxyphyllum* and *G*. *americanum* were active against Gram-positive bacteria tested including *Staphylococcus aureus* (MIC = 2.5 mg/mL and 5 mg/mL), *Enterococcus faecalis* (MIC = 5 mg/mL and 5 mg/mL), *Streptococcus pneumoniae* (MIC = 2.5 mg/mL and 1.2 mg/mL), and *Streptococcus pyogenes* (MIC = 2.5 mg/mL and 1.2 mg/mL). The methanol extract from *G*. *oxyphyllum*, and the hexanic and chloroformic extracts from *G*. *hirsutum*, all of which were active against *S*. *aureus* (MIC = 5 mg/mL, 2.5 mg/mL, and 5 mg/mL), *S*. *pneumoniae* (MIC = 1.2 mg/mL, 1.2 mg/mL, and 2.5 mg/mL), and *S*. *pyogenes* (MIC = 2.5 mg/mL, 2.5 mg/mL, and 2.5 mg/mL). The methanolic extracts from *G*. *americanum* and *G*. *hirsutum* were active against *S*. *pneumonia* (MIC = 2.5 mg/mL and 2.5 mg/mL) and *S*. *pyogenes* (MIC = 2.5 mg/mL and 2.5 mg/mL), the hexane extract from *G*. *oxyphylum* was active against *S*. *pneumoniae* (MIC = 2.5 mg/mL) and *S. aureus* (MIC = 2.5 mg/mL) [53]. The hexane extracts, either flowers or leaves, of *G. oxyphyllum*, *G. liebmannii*, and *G. viscosum* showed inhibition against both *S*. *aureus* and *Bacillus cereus*, except the *G. viscosum* leaves extract, which only inhibited *B*. *cereus*. The hexane extract of the flowers of *G. oxyphyllum* inhibited both *E*. *coli* and *Salmonella typhimurium*, and the hexane extract of leaves showed activities against *S*. *typhimurium* [13]. The essential oil from *G. affine* exhibited a potent inhibitory effect against the bacteria (*E*. *coli*, *S*. *aureus*, *Bacillus subtilis*, *B*. *cereus*, *Bacillus laterosporus laubach*, and *Salmonella typhimurium* with MIC values of 1.56 ± 0.41 μg/mL, 0.39 ± 0.12 μg/mL, 0.78 ± 0.30 μg/mL, 0.78 ± 0.11 μg/mL, 0.78 ± 0.13 μg/mL, and 0.78 ± 0.19 μg/mL, respectively), yeast (*Saccharomyces cerevisiae* with MIC value of 0.20 ± 0.21 μg/mL), and fungi (*Aspergillus niger*, *Pencicillium citrinum*, *Rhizopus oryzae*, and *Aspergillus flavus* with MIC values of 0.20 ± 0.18 μg/mL, 0.20 ± 0.15 μg/mL, 0.20 ± 0.19 μg/mL, and 0.20 ± 0.20 μg/mL, repectively) [52]. The methanol extract of aerial parts of *G*. *gaudichaudianum* showed activities against two subcutaneous fungi *Fonsecaea pedrosoi* at MIC 12.5 μg/mL and *Sporothrix schenckii* at MIC 50 μg/mL in the agar dilution assay [54].

### 3.3. Anti-Complementary Activity

Various chromatographic procedures on the ethyl acetate fraction of *G*. *affine* using silica gel, Sephadex LH-20, ODS, and MCI gel led to the isolation of 27 flavonoids. All compounds were evaluated for their anti-complementary activity on the classical pathway of the complement system in *vitro*, and some isolated flavonoids including the positive control heparin inhibited complement activation in a dose-dependent manner. Three caffeoyl flavone glycosides, apigenin 4'-*O*-*β*-d-(6''-*E*-caffeoyl)-glucopyranoside (**2**), apigenin 7-*O*-*β*-d-(6''-*E*-caffeoyl)-glucopyranoside (**3**), and luteolin 4'-*O*-*β*-d-(6''-*E*-caffeoyl)-glucopyranoside (**9**), and a caffeoyl flavonol glycosides, quercetin 4'-*O*-*β*-d-(6''-*E*-caffeoyl)-glucopyranoside (**34**), with the configuration of flavonoid-sugar-caffeoyl showed strong activity with IC_50_ values of 0.134 ± 0.016 mg/mL, 0.119 ± 0.013 mg/mL, 0.045 ± 0.005 mg/mL, and 0.147 ± 0.014 mg/mL, respectively. The anti-complementary activities of apigenin (**1**), luteolin (**6**), quercetin (**31**), quercetin 7-*O*-*β*-d-glucuronide (**34**), quercetagetin 7-*O*-*β*-d-glucopyranoside (**41**), and isorhamnetin (**42**) were moderate, and that of 6-hydroxyluteolin 7-*O*-*β*-d-glucopyranoside (**10**) was weak. The other compounds, luteolin 4'-*O*-*β*-d-glucopyranoside (**7**), acacetin 7-*O*-rutinoside (**16**), quercetin 4'-*O*-*β*-d-glucopyranoside (**32**), 5-hydroxy-3,6,7,8,4'-pentamethoxyflavone (**51**), 5,7-dihydroxy-3,8,4'-trimethoxyflavone (**59**), and 5,7-dihydroxy-3,8,3',4'-tetramethoxyflavone (**60**), did not show anti-complementary activity. A comparison of the chemical structures showed that the configuration of flavonoid-sugar-aromatic side chain can play an important role in structure-activity relationships, in which the types of flavonoids are less important. Additionally, there were no active compounds with a substituent on the 4'-position of the B ring in the oxygenated derivatives. It appears that the hydroxy group at the 4'-position in the flavonoid is essential for anti-complementary activity, but the activity is lost when it is substituted by methoxy group or sugar. However, the anti-complementary activity was also related to the number of hydroxylated groups [17].

### 3.4. Antitussive and Expectorant Activity

For thousands of years, the herb *G*. *affine* was decocted for treating respiratory diseases. The herbs of *G*. *affine* were extracted twice by the water decoction method for 1 h each time, and evaporation of the solvent gave a viscous material. The water extract (be equal to 18 g/kg, 12 g/kg, and 6 g/kg of plant material) was orally administrated to coughing mice induced by ammonium hydroxide and coughing guinea pigs induced by acitric acid, and mice injected with phenol red, respectively, to evaluate its potential expectorant and antitussive activity. The extract significantly prolonged the tussive delitescence and decreased the cough frequency caused by ammonium hydroxide and acitric acid, as well as the mucus secretion from mouse tracheas obviously increased by measuring the tracheal output of phenol red [55]. Campos-Bedolla *et al*. investigated the effect of methanol extract from *G*. *conoideum* on the responses to contractile agonists in guinea pig tracheas and the possible role of l-type Ca^2+^ channels in tracheal guinea pig isolated myocytes. Cumulative concentration-response curves to carbachol or histamine, as well as contractile responses to KCl were evaluated with or without 30 min preincubation with 20 or 100 μg/mL methanol extract, and intracellular Ca^2+^ concentrations were measured by microfluorometric method in isolated tracheal myocytes with or without preincubation with 0.1 μg/mL, 0.31 μg/mL, and 1 μg/mL methanol extract. The results showed that the extract significantly diminished the contractile responses to histamine, but not to carbachol or KCl, and significantly reduced the intracellular Ca^2+^ rise induced by 60 mM KCl in isolated myocytes. Because histamine contractile responses are largely dependent on extracellular Ca^2+^ and KCl responses are mainly mediated through l-type Ca^2+^ channels, the results suggested that methanol extract from *G*. *conoideum* might be acting as a partial blocker of these Ca^2+^ channels [10]. Hexane extract of *G*. *liebmannii* was the most active relaxant extract (IC_30_ = 54.23 ± 19.47 μg/mL with 99.5 ± 3.2% of relaxation) than dichloromethane extract (IC_30_ = 120.22 ± 5.27 μg/mL with 76.44 ± 2.3% of relaxation) and methanol extract (IC_30_ = 190.25 ± 30.02 μg/mL with 45.94 ± 10.3 % of relaxation) on guinea pig trachea smooth muscle. Hexane extract produced a parallel rightward shift of the concentration–response curve of carbachol (IC_50_ = 0.04 ± 0.0013 μM) in a competitive manner at concentrations of 177 μg/mL (IC_50_ = 0.20 ± 0.0089 μM) and 316 μg/mL (IC_50_ = 0.19 ± 0.001 μM), but did not modify the concentration–response curves for histamine (IC_50_ = 4.4 ± 0.36 μM) at concentrations of 100 μg/mL, 177 μg/mL, and 316 μg/mL. The relaxant effect of hexane extract (100μg/mL, 133μg/mL, 177 μg/mL, 237 μg/mL, and 316 μg/mL for block of ATP-sensitive potassium channel or 31 μg/mL, 100 μg/mL, 177 μg/mL, and 316 μg/mL for β-adrenergic receptors) of *G*. *liebmannii* was unaffected by the presence of propranolol or glibenclamide. However, hexane extract (87 μg/mL, 130 μg/mL, and 316 μg/mL) produced a leftward shift of the concentration-response curves of forskolin, nitroprusside, isoproterenol, and aminophylline, suggesting that *G*. *liebmannii* induced relaxation of the tracheal muscle, probably via phosphodiesterase inhibition [56]. By employing a bioassay-guided fractionation of the active hexane extract of *G*. *liebmannii*, using the model of isolated trachea from guinea pig, gnaphaliin A (**53**) and gnaphaliin B (**46**) were identified as the active relaxant compounds. Gnaphaliin A (EC_50_ = 195.97 ± 36.07 μM) and gnaphaliin B (EC_50_ = 134.04 ± 6.41 μM) showed more potent relaxant properties than aminophylline (EC_50_ = 534.50 ± 27.88 μM), a well-known relaxant drug can be used to treat bronchial asthma, chronic asthmatic bronchitis and chronic obstructive pulmonary disease [37].

### 3.5. Insect Antifeedant Activity

During the choice leaf-disk bioassay for evaluation of insect antifeedants, Morimoto, *et al*. initially recognized that the hexane and ether extracts of *G*. *affine* have potential antifeedant activity against a polyphagous insect, the common cutworm, *Spodoptera litura*. The flavonoids, 5-hydroxy-3,6,7,8-tetramethoxyflavone (**50**), 5-hydroxy-3,6,7,8,4'-pentamethoxyflavone (**51**), 5,6-dihydroxy-3,7-dimethoxyflavone (**61**), and 4,4',6'-trihydroxy-2'-methoxychalcone (**67**), have been isolated and have been evaluated for their antifeedant activity. There are significant relationships between insect antifeedant activity and the chemical structures of the flavonoids. Compound **50** and **61** were shown to have the strongest insect antifeedant activities. On the other hand, compound **51** was found to have less activity than the previous two flavonoids. These flavonoids were polymethylated, excluding the phenol by hydrogen bonding with the carbonyl group at the 5-position of the flavones, **50** and **61**, especially, have only a hydrogen substituent on the B-ring. Based on the bioassay evaluation, introduction of a methyl ether on the B-ring of the flavonoid decreases the insect antifeedant activity. In comparison, the chalcone **67** had a weaker activity [41].

### 3.6. Cytotoxic Activity

The compound 5,7-dihydroxy-3,6,8-trimethoxyflavone (**58**) derived from *G*. *elegans* effectively decreased cell viability of human colon cancer Caco-2 cells (EC_50_ = 12.42 μM), human pancreatic cancer Panc28 cells (EC_50_ = 51.76 μM), and human colon cancer HCT-116 cells (EC_50_ = 69.99 μM) in a concentration-dependant manner. However, that failed to decreased cell viability of human pancreatic cancer MIA PaCa cells, human breast cancer MCF-7 and SK-BR3 cells, and human prostate carcinoma LNCaP and PC3 cells. Cell viability of normal colon fibroblasts CCD-112 coN was unaffected after a 24 h treatment with increasing concentrations of the compound between 5–80 mM [16]. Compound 5,7,3',4'-tetrahydroxy-3-methoxyflavone (**64**) isolated from the Mexican plant *G*. *indicum* inhibited phorbol ester tumor promoter-enhanced phospholipid synthesis and sugar transport activity in cultured cells [23].

### 3.7. Anti-Inflammatory Activity

The air-dried flowers of *G. stramineum* were successively extracted with *n*-hexane, methanol, and water. Each extract was tested orally for anti-inflammatory activity using carrageenan-induced edema in rat paws. The methanol extract (140 mg/kg) was the most active, displaying 36.8% inhibition of edema (5 h), while the *n*-hexane extract (200 mg/kg), inhibiting the edema by 35.7%, was less active. The aqueous extract did not show significant anti-inflammatory activity. Bioassay-guided fractionation of the methanol extract of *G. stramineum* resulted in the isolation of four caffeoylquinic acid derivatives, 4-*O*-caffeoylquinic, 3,5-di-*O*-caffeoylquinic, 4,5-di-*O*-caffeoylquinic, and 3,4,5-tri-*O*-caffeoylquinic acids (**111** and **113**–**115**, resp.), and quercetin glycosides, isoquercitrin, quercetin 3-*O*-*β*-d-galactopyranoside, and rutin (**36**–**38**, resp.). Caffeoylquinic acid derivatives were tested in activated human macrophages for their activities on some human leukocyte functions related to inflammatory mechanism such as on monocyte migration and superoxide anion production. Compounds **113** and **114** exhibited an appreciable anti-inflammatory activity, while compound **115** was inactive. Compound **113** inhibited the peak of chemotactic index at a concentration of 1 × 10^−11^ M, but revealed a significant activity at a concentration as low as 1 × 10^−13^ M; compound **114** blocked the chemotaxis only at a concentration of 1 × 10^−7^ M; compound **115** was completely at any of the tested concentration (1 × 10^−7^ M–1 × 10^−17^ M). Quercetin glycosides (glucoside, galactoside and rutinoside) were able to reduce the edema induced by carrageenan and the exudative response induced by cotton pellet granuloma. The comprehensive evaluation showed that the anti-inflammatory activities of the extracts of *G. stramineum* may be due to a combination of caffeoylquinic acid derivatives and flavonol glycosides [36].

### 3.8. Hypoglycemic Activity

The decoction of *G*. *uliginosum* was documented to reduce experimental epinephrine and diabetic hyperglycemia but not to elevate the decreased blood insulin level in an epinephrine hyperglycemia model and alloxan diabetes in rats and mice [57]. In 1995, Tachibana, *et al*. found that the ethyl acetate and methanol extracts of *G*. *affine* displayed inhibitory effects on aldose reductase (IC_50_ = 4.3 μg/mL and 1.4 μg/mL, respectively). Bioassay-guided fractionation resulted in the isolation of luteolin (**6**), quercetin (**31**), gnaphalin (**68**), and scopoletin (**121**). The aldose reductase inhibitory assay test revealed that compounds **6**, **31**, and **68** exhibited potent activities with IC_50_ values of 0.7 μM, 2.6 μM, and 4.5 μM, respectively, whereas compound **121** was less active [46].

### 3.9. Antihypouricemic Activity

The hypouricemic actions of *G*. *affine* was *in vivo* examined using oxonate-induced hyperuricemic mice. The water extracts of *G*. *affine* at 25, 12.5, and 6.5 g/kg injected intraperitoneally were demonstrated to possess potent hypouricemic effects [58].

### 3.10. Other Activities

The water extract of *G*. *affine* (2.0 g/kg, 1.0 g/kg, and 0.5 g/kg) exhibited the protective effect for carbon tetrachloride-induced acute liver injury [59]. A gel formulation containing the extract from *G*. *uniflorum* proved to afford a significant in vivo protection against UV-B-induced skin erythema in healthy human volunteers [32]. As reported by Kubo et al., the methanol extract of *G*. *cheiranthifolium* showed significant (<200 μg/mL) inhibitory activity for the oxidation of L-3,4-dihydroxyphenylalanine mediated by mushroom tyrosinase. The compounds luteolin 4'-*O*-*β*-d-glucopyranoside (7) and gnaphalin (**68**) exhibited significant (almost 100% at 100 μg/mL) inhibition of the oxidation of L-3,4-dihydroxyphenylalanine by tyrosinase. Their limited availability prevented further study, so neither ID_50_ values nor their mode of inhibition were investigated. [25]. The ethyl acetate extract of *G*. *affine* promoted the rabbit platelet aggregation induced by ADP, whereas its methanol extract inhibited the aggregation. Compound **68** isolated from this plant showed an inhibitory effect (IC_50_ = 1.6 mM) on the rabbit platelet aggregation induced by PAF of about the same potency as that of quercetin (IC_50_ = 1.7 mM) [47].

## 4. Conclusions

Plants of the genus *Gnaphalium* are widely distribute all over the World, and many species are traditionally used as wild vegetables and inh folk medicine. In this review, we summarized the secondary metabolites reported from *Gnaphalium* species, as well as their biological activities. From our review, it can be concluded that phytochemistry investigations mainly focused on *ca*. 31 species. With regard to the 200 species of this genus, there are still many species that have received little or no attention. Further studies to exploit phytochemical constituents and biological activities from the plant of this genus are necessary to develop more potentially value-added products used in food and pharmaceutical industry.
